# Endourological Treatment of Hydrocalycosis in a Patient With Kidney Transplantation

**DOI:** 10.7759/cureus.64597

**Published:** 2024-07-15

**Authors:** Konstantinos Douroumis, Panagiotis Katsikatsos, Konstantinos Kotrotsios, Napoleon Moulavasilis, Evangelos Fragkiadis, Konstantinos Stravodimos, Dionysios Mitropoulos

**Affiliations:** 1 First Department of Urology, National and Kapodistrian University of Athens School of Medicine, Athens, GRC

**Keywords:** transplant biopsy, hydrocalycosis, balloon dilation, endourology, transplanted kidney

## Abstract

Hydrocalyx is the obstruction of a renal calyx resulting from infundibulopelvic stenosis or diminution and can be congenital or acquired. A 37-year-old man with a history of preemptive kidney transplantation in 2007 and transplant rejection underwent another ABO-incompatible transplant. During follow-up four months after transplantation, a transplant biopsy was performed, which revealed acute thrombotic microangiopathy. Seven months after transplantation, the patient was admitted to the hospital because of elevated creatinine levels and dilatation of the upper calyx on ultrasound examination. Upper calyx hydrocalycosis and calyceal neck stenosis were diagnosed. Nephrostomy placement along with an antegrade double-J stent through the upper major calyceal neck was performed. Endoscopic dilatation of the narrowed neck of the upper major calyx 10 days after hydrocalyx decompression was performed without intraoperative or postoperative complications. During follow-up, the patient was asymptomatic, had steady creatinine levels, and showed no signs of obstruction on ultrasound. This case highlights that treatment with balloon dilation of the calyceal neck appears to be an effective solution that respects the renal parenchyma and function.

## Introduction

Hydrocalyx is defined as the obstruction of a renal calyx, resulting from infundibulopelvic stenosis or diminution [1]. This condition was first described by Rayer in his “Traite des Maladies des Reins” in 1841, but K.H. Watkins and Winsbury White were the first to use the term hydrocalycosis in 1939 [2]. These can be congenital or acquired. When congenital, it is theorized to be a part of a spectrum of congenital obstructive dysmorphisms, known as infundibulopelvic dysgenesis [1].

The symptoms include flank pain, infection, pyocalycosis, hematuria, stone formation, and renal impairment. Its clinical presentation is variable and, in many cases, misleading. Differential diagnoses include renal cyst, caliceal diverticulum, and Fraley syndrome [3].

In asymptomatic patients, without significant danger of renal damage, there is no need for treatment, and observation is indicated [3]. In all other cases, surgical intervention is indicated and aims to improve drainage of the hydrocalyx [1,4,5]. Open surgical approaches such as infundibuloplasty, calicocalicostomies, and ureterocalicostomies have been described [1]. Partial nephrectomy or polectomy has also been advocated in cases of pyocalycosis. The evolution of endoscopic surgery has helped in the management of this entity, as dilatation with a balloon and incision of the stenosis with the laser have been described [6,7].

We present a case of acquired hydrocalycosis in a kidney-transplanted patient and the management with balloon dilatation of the calyceal neck stenosis.

## Case presentation

A 37-year-old man, with a history of preemptive kidney transplantation in 2007 and transplant rejection, was transplanted again with an ABO-incompatible transplant. The patient’s personal history included sensorineural hearing loss, hypertension, hyperthyroidism, and osteoporosis. The cause of renal impairment was unknown.

During follow-up four months after transplantation, a transplant biopsy was performed because of a baseline post-transplant creatinine of 3.94 mg/dL. Histopathology was remarkable for acute thrombotic microangiopathy (possible acute antibody-mediated rejection). CT renal angiography was unremarkable.

**Figure 1:**
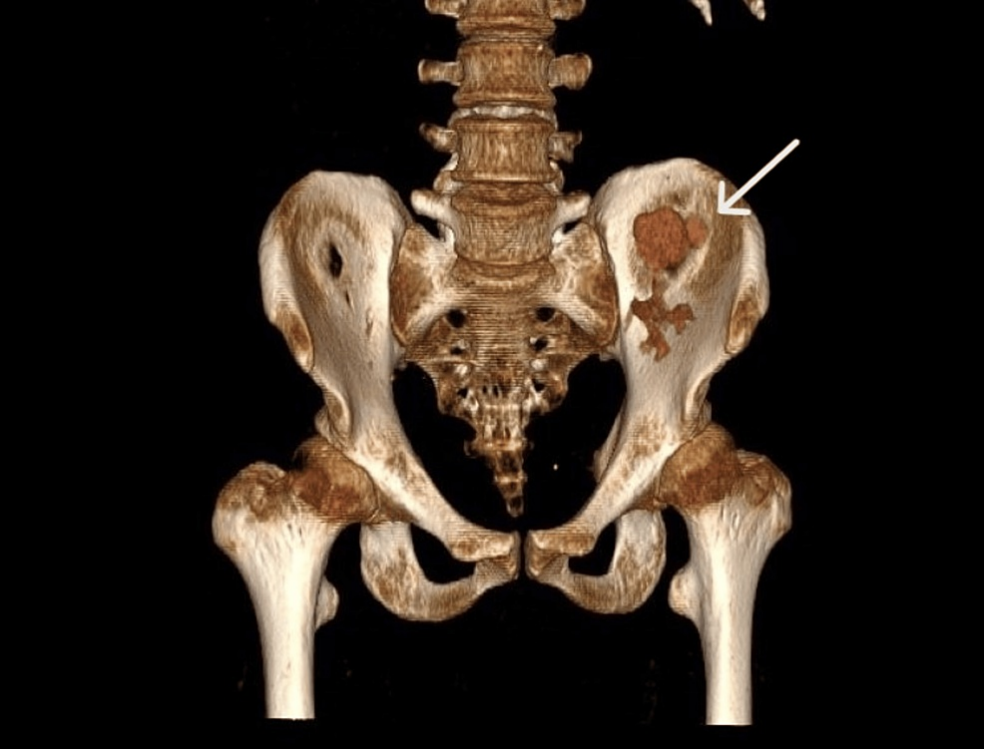
CT urography showing the dilatated upper calyx.

Seven months after transplantation, the patient was admitted to the hospital because of elevated creatinine (5.04 mg/dL) and dilatation of the upper calyx on ultrasound examination. CT urography was performed, and a diagnosis of upper calyx hydrocalycosis and calyceal neck stenosis was made (Figure 1). The urine analysis results were normal.

Because of acute renal failure and the need for hemodialysis, placement of a nephrostomy tube on the dilated calyx was determined to be the best solution. A nephrostomy placement along with antegrade double-J stent, through the upper major calyceal neck, placement was performed. The nephrostomy was closed but not removed. After hydrocalyx decompression, creatinine levels fell to 2.88 mg/dL.

The need for a six-month replacement of the double-J stent on a transplanted kidney, with all its possible complications, led us to find a more permanent solution without the need for stenting. We planned endoscopic dilatation of the narrowed neck of the upper major calyx 10 days after the hydrocalyx decompression.

**Figure 2:**
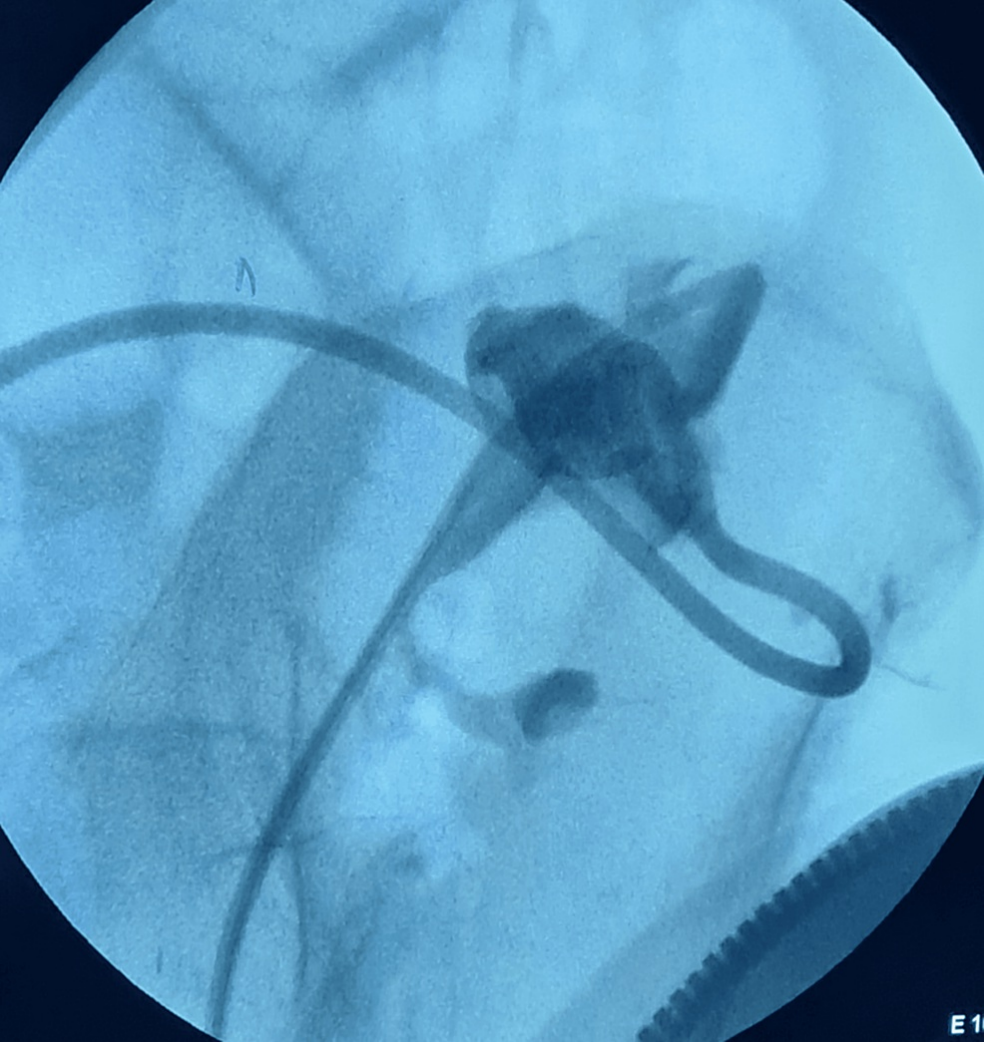
Intraoperative radioscopic image showing the curved glide wire (Terumo™) passed through the double-J stent to the hydrocalyx.

The first step involved passing a curved glide wire (Terumo™) through the double-J stent to the hydrocalyx (Figure 2). A UroMax Ultra™ High-Pressure Balloon (Boston Scientific™) 15 Fr × 6 cm was inserted over the wire, and dilatation of the calyceal neck was performed. A double-J stent 6 Fr × 26 cm was placed with its upper end coiled inside the hydrocalyx and its lower end coiled in the bladder. The patient had a normal postoperative course.

Nephrostomy was kept for safety, but it was kept closed. After 14 days, the nephrostomy tube was removed because the patient’s creatinine levels were stable and there were no signs of leakage from the nephrostomy. Three months after dilatation, retrograde pyelography was performed without any signs of stenosis, and the double-J stent was removed. During follow-up (24 months), the patient was asymptomatic, had stable creatinine levels (2.9 mg/dL), and showed no signs of obstruction on ultrasound.

## Discussion

Hydrocalycosis can be difficult to diagnose, as its symptoms are uncharacteristic and, in many cases, misleading. When a dilated calyx is discovered on ultrasound examination, renal cysts, caliceal diverticulum, and Fraley syndrome must be excluded. The examination of choice for differential diagnosis is CT urography, which has become the mainstay of urinary tract abnormality imaging. The aforementioned entities have specific characteristics on CT urography; more specifically, a simple renal cyst does not communicate with the calyceal system. In the case of a calyceal diverticulum, no renal pyramid or papilla is observed, whereas, in the context of Fraley syndrome, upper pole dilatation secondary to a vascular impression on the superior infundibulum could be present [8].

In this case, renal impairment led to the patient’s workup. It is important to note the effect that a partial obstruction can have on a solitary kidney. On pretransplant testing, the donor did not show any signs of hydrocalycosis. Therefore, it was interesting to determine the cause of the calyceal neck stenosis. As the only intervention that was performed after transplantation was renal biopsy, we hypothesized that the stenosis was caused by the biopsy. Another fact that supports this hypothesis is that CT renal angiography performed one day before the biopsy showed no evidence of obstruction. However, no prior cases of renal biopsy-induced infundibular stenosis have been reported.

Treatment options include open surgery (infundibuloplasty, calicocalicostomy, ureterocalicostomy) [1], open and laparoscopic excision [5], and an endourological approach. Open surgical treatment was excluded because it could significantly impair the transplanted renal parenchyma. The laparoscopic approach was also rejected due to extensive fulguration and arterial vasculature clamping, which led to parenchymal loss and ischemic injury, respectively, and, eventually, to possible deterioration of the transplanted kidney function [5,9]. Although less destructive laparoscopic approaches without vascular clamping have been described, we decided not to jeopardize the functional integrity of the solitary kidney [10].

Considering these, we opted for an endourological approach as it is less traumatic than open approaches and results in less renal parenchymal damage. Several endourological techniques have been proposed, with the cold knife infundibular incision being the first [11,12]. There have been reported cases of endourological treatment with balloon dilatation and laser incision of the stenosis using holmium (Ho):YAG laser [6] and a thulium fiber laser [7].

After dilatation and incision of the stenosis, these patients were symptom-free, without the need for stenting. However, less promising results emerged from a series of patients with infundibular stenosis, where one of the two patients treated with Ho:YAG laser combined with balloon dilatation, electrocautery, and stent placement experienced recurrence of the stenosis after three months [13]. As our patient had mild renal impairment and a solitary kidney, balloon dilation was performed without incision of the stenosis to minimize the potential loss of the renal parenchyma.

## Conclusions

Hydrocalyx is a rare entity, and its diagnosis may be challenging. The absence of symptoms and an essentially normal renal function can justify conservative management with observation. We describe the case of a transplanted patient with a dilatated upper major calyx. This hydrocalycosis appears to have been caused as the result of the upper major calyceal neck stenosis after a renal biopsy. The already impaired renal function along with the fragility of a solitary transplanted kidney made a minimally destructive surgical intervention necessary. The endourological treatment with balloon dilatation of the calyceal neck, with adjuvant incision, seems to be an effective solution, which respects the renal parenchyma and function.

## References

[1] Bayne CE, Peters CA (2016). Congenital infundibulopelvic stenosis: indications for intervention, surgical technique, and review of literature. J Pediatr Urol.

[2] Wilhelmi OJ (1949). Hydrocalycosis. J Urol.

[3] Dzefi-Tettey K, Mensah YB, Kyei JM, Gbadamosi H, Kyei MY (2021). Hydrocalyx presenting as lumbar pain. A case report and review of the literature. Radiol Case Rep.

[4] Atakan IH, Pekindil G, Alagöl B, Inci O (2000). A new cause of curvilinear renal calcification: calcified hydrocalycosis. Eur J Radiol.

[5] Wolf JS Jr (2000). Caliceal diverticulum and hydrocalyx. Laparoscopic management. Urol Clin North Am.

[6] Kim HL, Gerber GS (2000). Use of ureteroscopy and holmium:yttrium-aluminum-garnet laser in the treatment of an infundibular stenosis. Urology.

[7] Sharma N, Sengupta P, Shelmire L (2022). Large hydrocalyx mimicking as renal cyst and treated by Thulium fiber laser infundibulotomy. Urol Case Rep.

[8] El-Ghar M, Refaie H, Sharaf D, El-Diasty T (2014). Diagnosing urinary tract abnormalities: intravenous urography or CT urography?. Rep in Med Imaging.

[9] Porpiglia F, Bertolo R, Checcucci E, Amparore D, Manfredi M, Fiori C (2018). Laparoscopic nephron-sparing calycectomy for treating Fraley's syndrome. Urol Int.

[10] Armstrong JM, Soni SD, Link RE (2016). Laparoscopic nephron-sparing treatment of upper pole infundibular obstruction due to Fraley's syndrome. Urol Case Rep.

[11] Schneider AW, Conrad S, Busch R, Otto U (1991). The cold-knife technique for endourological management of stenoses in the upper urinary tract. J Urol.

[12] Zattoni F, Tasca A, Milani C (1990). [Endo-urology in diseases of the upper urinary tract other than tumors and calculi]. Ann Urol (Paris).

[13] Kieran K, Nelson CP, Wolf JS Jr (2006). Ureteroscopy for symptomatic hydrocalices: a case series. J Endourol.

